# Symptom Checklist-90-Revised: A structural examination in relation to family functioning

**DOI:** 10.1371/journal.pone.0247902

**Published:** 2021-03-12

**Authors:** Rapson Gomez, Vasileios Stavropoulos, Daniel Zarate, Olympia Palikara

**Affiliations:** 1 Federation University, Ballarat, Victoria, Australia; 2 National & Kapodistrian University of Athens, Athens, Greece; 3 Victoria University, Melbourne, Victoria, Australia; 4 University of Warwick, Coventry, United Kingdom; International Telematic University Uninettuno, ITALY

## Abstract

The accurate assessment of psychopathological behaviours of adolescents and young adults is imperative. Symptom Checklist-90-Revised (SCL-90-R) is one of the most comprehensive and widely used scales addressing this purpose internationally. Interestingly, associations between the different SCL-90 symptoms and family functioning have been highlighted. Nevertheless, the scale’s factorial structure has often been challenged. To contribute in this area, this study scrutinizes the psychopathological dimensions of the Symptom Checklist-90-Revised (SCL-90-R) in a large cohort of high school students (Mean age = 16.16; SD = .911) from Greece. It addresses this aim by: a) using first order and bi-factor confirmatory factor analysis, and exploratory structural equation models and; b) investigating the factors’ associations with family functioning. A total of 2090 public Greek High School students completed the SCL-90-R and the Family Adaptability and Cohesion Scale IV (FACES-IV) covering family functioning, satisfaction and communication. Six different solutions, yielded by separate permutations of CFA, ESEM, and bifactor models, were evaluated. Based on global fit, the clarity, reliabilities and the family functioning links of the dimensions in the models, the ESEM oblique model with the theorized nine factors emerged as the optimum. This model had adequate fit, and symptom dimensions were well defined. Also six of the nine factors demonstrated external associations with family functioning, satisfaction and communication. The clinical assessment benefits of these results are discussed.

## Introduction

The Symptom Checklist-90-Revised [1,2] is a widely used self-report questionnaire for measuring a range of psychological and psychiatric symptoms. It involves nine primary symptom dimensions, which entail somatization (SOM, for distress related to one’s body/physiological experiences), obsessive-compulsive (O-C, for intrusive thoughts and compulsive actions), interpersonal sensitivity (IS describing self-perceived inadequacy/inferiority in relationships with others), depression (DEP, for low mood and decreased sense of meaning), anxiety (ANX, for anxious symptoms and experienced tensions), hostility (HOS, for aggressiveness towards others), phobic anxiety (PANX, for fears related to specific stimuli), paranoid ideation (PI, for projections to others and persecutory cognitions), and psychoticism (PSY, for psychotic and schizophrenic behaviours; [1,2]). The instrument aims to contribute to accurately assessing one’s subjective (e.g. not clinically obserbable) experience of psychopathology, without requiring the involvement of mental health professionals (i.e. saving resources; [3]). Derogatis, Lipman, Rickels, Uhlenhuth and Covi (1974) [4] first introduced the Hopkins Symptom Checklist (HSCL) to self-assess the subjective experience of SOM, O-C, IS, DEP, and ANX symptoms. The five HSCL symptom categories were expanded with the addition of HOS, PANX, PI, and PSY by Derogatis, Lipman and Covi (1973) [5], resulting to the Symptom Check List 90 items (SCL-90). In 1994, Derogatis revised the SCL-90 to amend/replace psychometrically underperforming ANX and O-C items. Furthermore, three global distress measures and seven general items were added, informing the current version of the SCL-90-R [3].

The scale has been extensively used internationally, as well as in Greece, where the sample of the current study comes from, to assess diverse clinical, forensic and community populations across various age ranges [3,6]. Indicatively, the SCL-90-R has been successfully employed for the psychopathological examination of: a) female adolescents presenting with binge eating disorder, anorexia and bulimia nervosa and their parents [7]; b) early adolescents, who had lost a parent within their first years of life, and their surviving parents [7]; c) adult eating disorder (ED) diagnosed clients attending outpatient treatment programs [8]; d) adult substance use disorder patients [9,10]; e) impulse control disorder patients [9] and; f) adult incarcerated offenders [11]. In that context, the SCL-90-R subscales present to be of particular relevance regarding the psychopathological symptoms of adolescents and young adults [12]. Preti and colleagues (2019) [12] argue that the majority of mental healths symptoms first present during these years. Additionally, prefatory behaviours of major and life-long disorders, such as schizofreneia and bipolar disorder, although maybe not at a diagnosable level, tend to emerge the same period [13]. Developmental challenges, such as the intergration of one’s identity and life-orientation, their transition from more limited child responsibilities to adult independence and likely contradicting influences/messages received by peers, family and social institutions (e.g. church) have been implicated with higher psychological vulnerability for adolescents and emergent adults [14]. Thus, timely comprehensive assessment, such as that offered via the SCL-90-R, could be crucial for the containement/prevention of future psychopathological escalations in these populations.

### SCL-90-R and family functioning

Interestingly, the presence of psychopathology in adolescent and early adult populations has been disctinctively associated with one’s family functioning [7,15–23]. Literature has hypothesized that such associations may be explained bi-directionally [24]. Specifically, dysfunctional family relationships may precipitate and perpeatuate reactive psychopathological manifestations [24]. For instance, chaotic family boundaries and emotional distance between family members may invite (and/or accommodate pre-existing) depressive or anxious behaviours [25]. On the other hand, evidence also suggests that the presence of impactful psychopathological presentations by an off-spring, may also trigger (and/or exacerbate pre-existing) family dysfunctions [26]. Likewise, aggression and hostility by a young adult may compromise the level of communication within their family or the level of satisfaction experienced within one’s family [27].

To depict such associations, Tafà and colleagues (2017) [7] studied a sample of female adolescents presenting with ED and their parents. They used the SCL-90-R to measure their psychopathology and the Family Adaptability and Cohesion Evaluation Scale IV edition (FACES-IV) for their perceived family functioning [28]. The latter assumes that family functioning can be described by two main dimensions, flexibility and cohesion. Flexibility refers to the level of change in roles and rules (e.g. leadership), which is necessary to enable families to grow (e.g. progressively offsprings acquire more responsibilities). Although a moderate/balanced level of flexibility is deemed necessary for a family to achieve changes without compromising its stability, too low levels may reflect rigidity. This does not enable the family as a whole and/or the family members independently to evolve. Similalry, excessive flexibility induces chaos, which undermines a family’s sense of stability. In that line, cohesion refers to the level of emotional bonding experienced between family members. A family with a balanced sense of cohesion allows its members to be connected/ interdependent without violating their independency. Nonethelss, too high cohesion may eventuate a state of enmeshment, which deprives members from their independence. Similarly, too low levels of cohesion may invite a state of disengagement between members, which reduces their sense of connection. Enmeshment and disengagement can also compromise family functioning hosting forms of psychopathology, especially when combined with either rigidity or chaos [28]. Olson and colleagues (2006) [28] additionally suggest that balanced flexibility and cohesion are correlated with better communication between family members and a sense of satisfaction regarding one’s family. Indeed, the work of Tafà and colleagues (2017) [7] confirmed that ED adolescents and their parents have distinctive psychopathological profiles, whilts their family functioning patterns may predict different forms of comorbid psychopathology. For instance, anorexic adolescents presented more anxious, depressed, hostile and obsessive-compulsive compared to their bulimic and binge-disorder counterparts. Interestingly, when considering one’s family functioning, higher levels of hostility linked with higher levels of family rigidity among anorexic adolescents. Finally, higher reported levels of somatization tied with higher levels of family enmeshment among bulimic adolescents.

Despite these, there is a dearth of evidence comprehensively examining a variety of different symptoms (such as those described in SCL-90-R) and different family functioning aspects using a large cohort of young people [29]. Such findings would be important for guiding the implementation of evidence-based prevention and intervention programs. This could be achieved by selectively targeting either different types of families or specific family functioning aspects such as flexibility (i.e. family boundaries’ tightness) and cohesion (i.e. emotional distancing between family members) to address certain types of symptoms [30]. This study aims to answer such questions by examining the associations of the different dimensions of psychopathological symptoms of the SCL-90-R with aspects of family functioning in a large and normative cohort of Greek adolescents and young adults.

### SCL-90-R structure

For such research to be effectively implemented, the structure of the SCL-90-R and its included sub-dimensions need to be first established. Nevertheless, these have been challenged via the use of different analytical methods [31–35]. First, exploratory procedures such as principal component analysis (PCA) and exploratory factor analysis (EFA) have been used to examine the SCL-90-R symptoms dimensions [12,31,32,36]. Such methods allow for cross-loadings that may compromise the clarity of the factors when inter-item associations occur [37]. Indeed, the initial SCL-90 PCA study by Derogatis and Cleary (1977) [38] showed that the PSY factor was not well defined, whilst ANX and PANX items overlapped. Cyr and colleagues (1985) [36] also supported that the nine dimensional structure of the SCL-90-R is lacking stability due to multiple cross-loadings. Subsequent PCA and EFA studies have generally supported these findings since then [39–42].

#### Confirmatory procedures

These highlight the importance of implementation of confirmatory factor analysis (CFA) to study the SCL-90-R structure [31,33–35]. In CFA items link exclusively to their designated dimensions, and cross-loadings are not allowed [43,44]. Interestingly, CFA studies of the SCL-90-R have supported at least five different possible structures: a) uni-dimensional (i.e. all items loading on a single general psychopathology factor); b) nine first-order factors (i.e. nine factors directly related to the items without any overarching/inclusive dimension); c) the nine-dimensional oblique (i.e. assuming some correlation between the nine dimensions); d) the higher order dimensional structure (the nine primary dimensions linking on one higher order general dimension) and; e) the bi-factor structure (all items linking with one general dimension and concurrently with their own nine specific factors [9,12,31–36].

Among these models, a number of past CFA literature has shown better global fit for the bi-factor model [9,12]. As bi-factor CFA models are more flexible in accommodating different response patterns, they tend by definition to fit better [45]. Nonetheless, this requires further investigation for several compelling reasons. Firstly, the bi-factor model has not always been supported. For example, Urbán et al. (2016) [32] favoured the bi-factor structure in only one of the two samples they examined. Secondly, the findings for bi-factor SCL-90 solutions have shown numerous cross-loadings [9], as well as negative loadings on designated dimensions [31]. Thirdly, although research has generally reported substantial reliability for the general factor [9,12,32,35], the specific factors have often not shown acceptable reliability [12,32,35].

In this context, CFA limitations have also been illustrated [46,47]. Specifically, the zero CFA cross-loadings may compromise the accurate interpretation of items in multidimensional measures (as is the case with the SCL-90-R). This is due to items often simultaneously providing information (at least to some degree) for dimensions other to their primary designated one [47]. This limitation refers also to the bi-factor CFA model, where the different dimensions are not inter-correlated (orthogonal model). Thus, the overall/general dimension captures all the shared information (i.e. variances) across the different items, whilst the sub-dimensions capture an amount of information which is distinct [47].

#### Exploratory equation modelling procedures

Relatedly, to date there is no robustly accepted factor model for SCL-90-R. Clearly more studies are needed in this respect [31,32]. However, more PCA, EFA, CFA, and Bifactor CFA (BCFA) studies are unlike to be fruitful. Alternatively, the application of proven superior modelling procedures that can counterbalance their limitations, such as exploratory structural equation modeling (ESEM) and bi-factor exploratory structural equation modeling (BESEM) could provide more reliable findings. This possibility is also reinforced by coceptual and theoretical arguments assuming that: a) psyhopathological symptoms are interrelated and; b) behavioural presentations of different diagnostic entities, such as those inquired in SCL-90, may be shared (i.e. sleep disturbanses common between anxiety and depression, [48]).

Asparouhov and Muthén (2009) [49] have advocated the implementation of exploratory structural equation modeling (ESEM) with targeted rotation, as being especially useful for assessing the structure of multi-dimensional scales. ESEM combines the positives of the EFA (i.e. enabling cross-loadings) and CFA (i.e. being conceptually driven, and allowing the examination of a pre-defined structure) approaches. Subsequently, in an ESEM structure, items link to their designated specific dimensions, as well as all other dimensions (at rates approximating zero). Studies have evidenced that this allows ESEM to be superior to the simple EFA and CFA approaches when testing factor structures [37,46]. Interestingly, the ESEM analysis with targeted rotation can be upgraded to a bi-factor ESEM (BESEM) analysis. This uniquely concentrates the positives of the CFA and EFA approaches, and also bi-factor modelling [37,47]. This is crucial for demistyfying the complex associations between the distinct and the overarching dimensions of psychopathological symptoms [48]. In the present study, such complex structures and associations are examined based on SCL-90-R ratings. Not surprisingly, these have been revealed to be multi-dimensional, with various cross-loadings, and entailing both general and specific psyhcopathological dimensions [12,32,35]. Despite these, to date, neither the ESEM nor BESEM approach have been employed for studying the factor structure of the SCL-90-R. It is additionally noted that even if the ESEM model or the BESEM model shows good global fit; it is still necessary for the dimensions in the model to be clearly defined [47].

### Current study

Taking these into consideration, the primary goal of the present research was to assess the dimensionality of the SCL-90-R using CFA, bi-factor CFA, ESEM and BESEM approaches. A large and normative cohort of adolescents and young adults from Greece was examined. Several different alternative structures were comparatively studied. These entailed: a) the CFA uni-dimensional; b) the first order CFA nine-dimensional oblique model (with primary dimensions for SOM, OC, IS, DEP, ANX, HOST, PANX, PI, and PSY); c) the higher order CFA model (with the nine primary dimensions linking on a single overarching psychopathological dimension); d) the BCFA structure (with one general dimension and specific sub-dimensions for SOM, OC, IS, DEP, ANX, HOST, PANX, PI, and PSY); e) the ESEM model (with dimensions for SOM, OC, IS, DEP, ANX, HOST, PANX, PI, and PSY); and f) the bi-factor ESEM (BESEM) structure (with one general dimension and specific dimensions for SOM, OC, IS, DEP, ANX, HOST, PANX, PI, and PSY). Model-based reliabilities (omega; [50]) for the various dimensions were also calculated in relation to the best fitting structure. In addition, external associations of the different psychopathological dimensions were examined in relation to family functioning aspects, as assessed with the FACES-IV scale, in line with Tafà and colleagues work (2017) [7].

## Research methods

### Subjects and procedure

The sample involved 2090 students of public Greek high schools in the broader Athens metropolitan area. The maximum estimated sampling error for this number of respondents is minus/plus 2.14% (95% confidence level; Z = 1.96)^1^. This satisfies the 4% requirement proposed (Hill, 1998) [51]. In addition, a conducted G-Power evaluation for a-priori estimations, linear multiple regression R^2^ deviation from 0, an effect size f^2^ = .15, an error probability of α = .05, power (1-β error probability) of .95, a non-centrality parameter λ = 63.60, a critical F of 1.30, an actual power of .951 and a number of 90 predictors, suggested that a minimum sample of 429 would be required [52]. This condition is also safely addressed here. Last, the current sample size is well above the rule of thump of minimum 20 participants per item considering EFA/CFA procedures (i.e. 20 x 90 = 1800 here; [53]). Participation, provided parents’ consent, exceeded 95% of approached population. Their average age was *M* = 16.16 (*SD* = 0.91). All students attending a public Greek high school were eligible to participate in the study. After-hours, vocational, public high schools were not exempted by the selection process. The presence/ absence of psychopathological symptoms was not assessed prior the study. Thus, students who likely presented with psychopathology were also not exempted. The mean (*SD*) age for males (49.7%) and females (50.3%) was 16.22 years (0.96) and 16.11 years (0.86), respectively. Although females and males differ significantly on age, *t* = 2.37, *p* < 0.05, this difference was of negligible effect size (Cohen’s *d* = .12).

Upon written approval of the Greek Ministry of Education, teacher and written parents’ consent (for the minors included in the sample) were secured (i.e. the parents/guardians of minors addressing the study sent letters of participation acceptance-or the opposite-to the school principal, who communicated the final participants’ list to the research team). Minors themselves had to finally orally aggree to participate. To encourage participation students were explained that if they did not partcipate in the study, they would need to attend the subjects taught duirng the time of the data collection. Data collection took place during class hours during the 2010–2012 school years. It is noted that access to students of public Greek high schools for research purposes, necessitates an initial permission from the research ethics committee of the pedagogical institute of the ministry of education (protocol number for the current data collection is 101359Γ2). A team of 13 specially trained researchers collected data in participants’ classrooms at either the beginning or end of school day. Participation was anonymous and students were not penalized if decided to discontinue.

### Measures

#### Family Adaptability and Cohesion Evaluation Scales IV (FACES IV)

Family relationships health/ well-being, satisfaction, and communication were measured using the Greek version [54] of the FACES IV [28]. The scale reflects the Circumplex Model of Marital and Family Systems [55]. It entails 42 items measuring family functioning in terms of two aspects: a) family cohesion and; b) family flexibility. In the Circumplex Model, cohesion and flexibility are viewed as curvi-linear, and thus very low and very high scores for both these aspects are deemed as maladaptive. In contrast, intermediate scores are considered balanced and adaptive [56]. Corresponding to this, FACES IV has six scales. They include two balanced sub-scales (Balanced Cohesion and Balanced Flexibility), two subs-cales measuring maladaptively low and high cohesion (Disengaged vs Enmeshed), and two sub-scales measuring maladaptively low and high flexibility (Rigid vs Chaotic). For all FACES IV items, responses ranged between 1 = *strongly disagree* to 5 = s*trongly agree*. Eelevated reported numbers on the two balanced sub-scales are refelective of more adaptive family relationships. Counterintuitively poorer family relationships are reflected by elevated scores in the four maladaptive sub-scales for cohesion and flexibility. For this study, the raw scores from each subscale informed the analyses.

For more robust and holistic evaluation, cohesion ratio, flexibility ratio, and total circumplex ratio scores are recommened to be calculated [28]. These more accurately reflect the relative amount of balance/unbalance for cohesion, flexibility, and the overall (circumplex) family functioning, respectively. Their values fluctuate from 1 to 10, with one indicating equal amount of balanced and unbalanced levels. Higher rates of suggest healthier family functioning [56].

The FACES IV Package also included the Family Communication Scale (FCS, [57]) and the Family Satisfaction Scale (FS, [58]). The FCS and the FSS (10 items each) assess levels of communication and satisfaction within the family, with responses from 1 =“strongly disagree” to 5 = “strongly agree” and higher scores indicating more positive performance across both aspects respectively. The FCS and FSS were additionally completed by the current sample. Like the FACES IV, the FCS and FSS are self-report measures that may be addressed by any family member above 12 years of age. For the current study, these measures were completed by the participants. The internal consistency (Cronbach α) for the 2 adaptive and the 4 maladaptive main FACES IV sub-scales in the current sample fluctuated from .60 to .71, while for the FCS and FSS these equaled 0.91.

#### The Symptom Checklist-90-R (SCL-90-R; [2])

Given the Greek speaking population, the Greek adaptation of the SCL-90-R was used in the study [59]. Further to the SCL-90-R information described in the introductory part, it is highlighted that different symptom dimensions are hypothesized to include unequal number of items (i.e. SOM, 12; O-C, 10; IS, 9; DEP, 13; ANX, 10; HOS, 6; PANX, 7; PI, 6; PSY 10). An item example is “Having thoughts that are not your own” (PSY subscale). Distinct items reflect different symptoms and are scored across 5-points (0 = “*not at a*ll” and 4 = “*very much*”), with more elevated numbers being indicative of more intense symptoms. For this study, the item scores were used as observable indicators in the CFA/ESEM analyses. The internal consistency (Cronbach’s α) values for the subscales in this data were SOM = .85; O-C = .79, IS = .81, DEP = .86, ANX = .85, HOS = .84, PANX = .73, PI = .74, and PSY = .78 and .97 for the general psychopathology factor.

### Statistical analysis

The Mplus software 7.3 [60] was employed to address all calculations. Given the argument made by Urbán et al. (2014) [31] that analytic methods for the SCL-90-R need to consider the nature of the ratings in this measure, the robust maximum likelihood estimator (MLR) was used. This estimator adjusts for violations of normality and is favoured for items with five or more answering points [60–62] such as those of SCL-90-R.

To examine which of the six different CFA (i.e. unidimensional; nine-dimensional oblique; higher order), BCFA, ESEM or the BESEM models was most applicable, a sequence of four successive steps was adopted. These entailed assessment of: a) global model fit criteria; b) dimension clarity; c) dimension reliability and; d) external associations envisaged with family functioning (as assessed with FACES-IV) based on the literature [15–22].

Therefore, we initially inspected the global fitness of each of the six models separately. Then we compared them to define those with the optimum fit. To assess the 6 models fit independently the Hu and Bentler (1999) [63] recommendations were followed. They proposed that RMSEA scores of .06, or lower reflect sufficient fit, .07 to .0158 moderate, .08 to .10 marginal, and >.10 poor fit. Regarding the CFI and TLI, rates of .95 and higher reflect sufficient fit, between .90 and .95 average, and less than .90 poor fit [63]. Next, comparative model evaluation for the nested models involved the estimation of the chi square difference (Δχ^2^, [37]). Provided the susceptibility for inflation that applies to χ^2^ and Δχ^2^ estimates, the current study additionally considered RMSEA and CFI discrepancies between models [60]. ΔCFI ≤ .010 and/or ΔRMSEA ≤ .015 were viewed as reflecting significant differences [64,65]. Adopting previous suggestions (e.g., [66,67]), we relied more on the differences in CFI values, given that these are less compromised by model complexity, sample size, and overall fit measurements.

Second, the two best fitting structures (as concluded in the first step) were evaluated. Their different dimension loadings/cross-loadings were assessed to confirm the quality/clarity of the dimensions. For this purpose, a rate of ≥.30 was adopted to indicate salience [68]. The model with the optimum fit based on the two initial steps was then chosen.

Third, we tested the omega coefficient (ω) reliabilities of the dimensions included in the optimum fit model [69]. The ω coefficient is understood as a saturation test that accounts for covariance among items in measuring scale reliability [50]. Bi-factor models employ omega hierarchical (ωh) for general factors and omega subscale (ωs) for specific factors [50,69]. The ω coefficient rates vary from 0 to 1 reflecting higher reliability as values increase [70]. The ratio of ωh to ω (called relative omega) indicates the proportion of variance in the overall score that is associated to the general dimension. Cut-off criteria for ωh, as determined by Reise, Bonifay, and Haviland (2013) [71], suggest a minimum value of .50 for acceptable scale reliability and a value of .75 for a more meaningful scale reliability. Furthermore, we used the standards recommended by Smits et al. (2014) [35] to classify all the ω values: substantial ≥: 30, moderate 20 to <: 30; and low <: 20.

Fourth, we also tested the external associations of the factors in the optimum fit structure with the FACES IV scores. Following suggestions outlined in Park et al. (2018) [72], FACES IV, FCS and FSS scale scores were regressed on to the SCL-90-R dimensions of the optimum model. Gender was included as a covariate in the regressions to control for confounding effects.

## Results

### SCL-90-R models fit

The global fit indices for the sequence of SLC-90-R alternative structures assessed are presented in Table 1. Following Hu and Bentler’s (1999) [63] proposals, for all the CFA models (e.g. unidimensional; nine-dimensional oblique; higher order and bi-factor), the RMSEA suggested good fit, whilst the CFI and TLI poor fit. For both the ESEM and BESEM structures, the RMSEA advocated good fit, while the CFI and TLI adequate fit. Furthermore, both these models showed better fit than all the other models, when the ΔCFI values (> .01) between all structure pairs were considered. Between the ESEM and the BESEM models, the AIC and BIC values were lower for the BESEM model. Thus, this was proposed as a better fitting structure than the ESEM one. However, there was no significant difference between these models in terms of ΔCFI and ΔRMSEA. Thus, it can be taken that when all the global fit values for these two models are considered together, the support for the ESEM and BESEM are somewhat comparable. Therefore, we then examined the clarity of the dimensions in both these models.

**Table 1:** Fit of all the models tested in the study.

	Fit Values					
Models	*χ*^2^ (*df*)	CFI	TLI	RMSEA (90% CI)	AIC	BIC
CFA 1-factor	15155.15 (3320)	.759	.743	.041 (.041 - .042)	473506	474911
CFA 9-factor	10749.53 (3284)	.842	.836	.033 (.032 - .034)	467584	469192
Second order CFA with 9 primary factors	11530.38 (3311)	.826	.821	.035 (.034 - .035)	468595	470050
BCFA with 9 specific factors	10978.58 (3237)	.836	.828	034 (.033 - .035)	467803	469676
ESEM 9-factor	5703.33 (2692)	.936	.919	.023 (.022 - .024)	461534	466482
BESEM with 9 specific factors	5298.41 (2618)	.943	.926	.022 (.021 - .023)	461093	466459

*Note*. CI = confidence interval; *χ*^2^ = chi-square; *df* = degrees of freedom; CFA = confirmatory factor analysis; ESEM = exploratory structural equation modeling; BCFA = bifactor confirmatory factor analysis; BESEM = bifactor exploratory structural equation modeling. RMSEA = root mean square error of approximation; CFI = comparative fit index; TLI = Tucker-Lewis Index; AIC = Akaike Information Criterion; BIC = Bayesian information criterion.

#### Dimensions’ clarity/factor loadings in the two best fitting models

Tables 2 and 3 presents the loadings for the dimensions of the ESEM and the BESEM structures. Table 4 provides an overview of the number of focused and non focused salient factor loadings in the ESEM and BESEM structures. As evident, most of the focused items associated significantly with their respective dimensions for the ESEM, with the exception of PI. Also the numbers of salient cross-loadings for all the different dimensions were low, ranging from 0 to 3, and there was no negative loading for any symptom. In contrast, relatively fewer items loaded on their BESEM specific dimensions. The majority of the symptoms for seven BESEM dimensions (OC, IS, DE, AN, PA PI, and PY) did not load saliently on their designated dimension. In addition, more salient loadings were present in the ESEM structure vs the BESEM. Although there were many significant cross-loadings in both these structures (ESEM & BESEM), these were relatively more significant in the ESEM. However, considering both models, only few cross-loadings were salient (ranging from 0 to 3 in the ESEM model, and 0 to 1 in the BESEM model). Also, while there were many significant negative cross-loadings across both structures, there were relatively more negative cross-loadings in the BESEM model. Additionally, the intercorrelations of all the SCL-90-R factors in the ESEM model ranged from .10 to .45. Conclusively, the ESEM dimensions were better defined than those of the BESEM. We therefore progressed in the examination of the two final steps of our procedure focusing on this structure.

**Table 2:** Standardized factor loadings in the SCL-90-R ESEM model.

	ESEM model with nine specific factors								
	SOM	OC	ÌS	DEP	AN	HOS	PANX	PI	PSY
Headaches (1)	**.43**								-.16
Faintness(4)	**.55**	-.14		.13					
Pains chest(12)	**.55**								
Pains lower back(27)	**.47**	.11							-.09
Nausea (40)	**.59**						.09		-.10
Soreness-muscles(42)	**.52**	.20							
Trouble breathing(48)	**.48**	-.13					.14		.14
Hot/cold spells(49)	**.40**				.11			.09	.10
Numbness(52)	**.48**	.15		-.13	.12				.10
Lump in throat(53)	**.42**								.14
Weakness-body(56)	**.58**	.18							
Heavy arms/legs(58)	**.45**							-.11	.18
Unpleasant thoughts(3)	.11	.*07*		**.37**		.16			-.11
Trouble remembering(9)	.24	**.30**	-.13		.19		.11		.11
Worried-sloppiness(10)		.20		.12	-.15	.15			
Feeling blocked(28)	.13	**.33**		**.35**	-.13				
Doing things slowly(38)		**.39**	.13						
Double-checking(45)	.03	**.43**	.14			-.07			
Difficulty deciding(46)		**.31**	.14	.19	.18				
Mind blank(51)	.19	.26		.15	.14				.17
Trouble concentrate (55)	.18	**.30**		.18		.15			
Repeating actions(65)		**.31**				.			
Critical of others(6)	-.10	.13	.*03*		.23	**.34**	.12		
Shy-opposite sex(21)		.12	**.35**	.12	-.16		.21		-.12
Easily hurt(34)	.07		**.30**	.25				**.31**	
Others unsympathetic(36)			**.30**	.19	.23	.09		**.47**	
Dislike(37)			**.41**					**.36**	.14
Inferior (41)	.13		**.43**	.14	-.14	-.13	.13		
Uneasy when watched(61)		.12	**.59**						
Self-conscious (69)			**.66**	.11				-.19	.11
Bother eating public(73)	.08		**.44**			.11		-.13	
Loss of sexual interest(5)				.*02*			.14		
Low energy/slow(14)	.10	.19		**.44**			.15		
Thoughts of ending life(15)		.09		**.48**					.16
Crying easily(20)	.19	-.16	.19	.19			.14	.17	-.21
Feeling trapped(22)				**.45**	.26				.13
Blaming yourself(26)		.14	.12	**.43**			-.11		
Feeling lonely(29)	.10	-.13	.17	**.50**	.22			.16	.18
Feeling blue(30)	.09	.05	.17	**.47**		.07			
Worrying too much(31)		.27	.11	**.36**					-.11
No interested (32)		.18	-.13	.*00*	.27	.22	.10		.18
Hopeless about future(54)	.10	.13		**.37**	-.14				.24
Everything is effort(71)	-.15	**.35**	.15	.21					
Feeling worthless(79)		.12	.21	**.32**	.11				.26
Nervousness(2)	.18			.29	.06	**.37**			-.14
Trembling(17)	**.37**			.	.15		.20		
Suddenly scared(23)				.26	.29	.08	.29		
Feeling fearful(33)				.17	.25		.38	.14	
Heart pounding(39)	**.43**	.11			.20	.08			
Feeling tense(57)	.21	.15			.11	**.41**			-.12
Spells of panic(72)					**.31**	.14	.26	-.15	.26
Can’t sit still (78)					.15	.23			.17
Bad is happening (80)	.11	.11			**.39**	.16			.09
Frightening thoughts(86)	.09	.09			.21	.10			.25
Easily annoyed(11)				.13		**.53**			-.21
Temper outbursts(24)						**.64**		.06	
Harm someone(63)				-.15	.13	**.66**			.16
Urges to break things(67)			.07			**.61**	-.12		.09
Arguing frequently(74)	.07	-.11			.12	**.68**		.10	
Shouting/throwing(81)	.06					**.62**			.09
Afraid on the street(13)					.14		**.50**		-.11
Afraid to go alone(25)					.13		**.57**		
Afraid- transport(47)	.14		.12	-.11			**.35**		
Avoid things (50)		.17					**.38**		.12
Uneasy in crowds(70)			**.44**		.14		**.30**	-.22	.16
Nervous when alone(75)							.26	.12	
Afraid faint in public(82)	**.32**				.26		.16		.17
Others are to blame(8)				.14	.21	.20		.19	
Can’t be trusted(18)		.09	.13	.13		.12		.24	
Feeling watched(43)	.09	.19	**.31**		.12		.13	.19	.10
Others not have beliefs(68)		.12	.16	.17		.18		.*05*	.17
Not getting credit(76)		.10	.17	.18		.13		.21	.14
Taken advantage of (83)		.09	.25					.18	.18
Thoughts being control(7)		.15			.15		.17		.*13*
Hearing voices(16)	.14					.13	.25		.22
Knowing thoughts(35)		.16						.24	.*01*
Thoughts not yours (62)		.17				.10			.21
Feeling lonely (77)		-.16		**.40**	.13				**.39**
Sexual thoughts (84)		.12	.12			.08			.19
Punished for your sins(85)		.20	.12						.19
Wrong-body(87)	.24	.08	.15		.25				.23
Never close to others(88)			.16	.19	.15				**.36**
Wrong-mind(90)	.10	.11		.12			.13		**.36**

Underlined factor loadings are targeted loadings. Italic factor loadings are nonsignificant targeted items. Boldfaced factor loadings are significant at *p* < .05. For correlations, ns = not significant ((*p > .05); *p < .05; all others correlations are significant at p < .001. G = Generral; SOM = Somatization; OC = Obsessive-Compulsive; IS = Interpersonal Sensitivity; DEP = Depression; AN = Anxiety; HOS = Hostility; PANX = Phobic Anxiety; PI = Paranoid Ideation; PSY = Psychoticism.

**Table 3:** Standardized factor loadings in the SCL-90-R BESEM model.

	Bifactor ESEM model with nine specific factors									
	G	SOM	OC	ÌS	DEP	AN	HOS	PANX	PI	PSY
Headaches (1)	**.38**	.29								
Faintness(4)	**.38**	**.43**	-.15							
Pains chest(12)	**.44**	**.40**		-.11						
Pains lower back(27)	**.41**	**.31**								
Nausea (40)	**.48**	**.44**						.07		-.08
Soreness-muscles(42)	**.44**	**.35**	.14							
Trouble breathing(48)	**.45**	**.36**	-.12					.11		
Hot/cold spells(49)	**.54**	.27		-.09						
Numbness(52)	**.51**	**.32**								
Lump in throat(53)	**.52**	**.30**				.10				
Weakness-body(56)	**.57**	**.38**	.12							
Heavy arms/legs(58)	**.51**	**.33**	.08							.12
Unpleasant thoughts(3)	**.61**		*-*.*08*					-.13		-.16
Trouble remembering(9)	**.36**	.16	.28						.12	
Worried-sloppiness(10)	**.31**		.17				.12		.13	
Feeling blocked(28)	**.56**		.22		.21					
Doing things slowly(38)	**.43**	-.13	.22							
Double-checking(45)	**.51**	-.08	.22				-.10			-.14
Difficulty deciding(46)	**.58**	-.09	.17				-.08			
Mind blank(51)	**.57**	.08	.22							.13
Trouble concentrate (55)	**.52**	.07	.26			-.17	.10			
Repeating actions(65)	**.43**		.*15*		-.16					
Critical of others(6)	.20	-.08	.12	.*03*			.26			
Shy-opposite sex(21)	**.41**	-.08		.23			-.09	.13		-.12
Easily hurt(34)	**.66**	-.09	-.23	*-*.*04*		-.21				
Others unsympathetic(36)	**.65**	-.15	-.12	*-*.*02*		-.26				
Dislike(37)	**.59**			.16					.32	
Inferior (41)	**.55**			**.31**			-.18		.11	
Uneasy when watched(61)	**.58**	-.10		**.35**			-.07			
Self-conscious (69)	**.58**	-.06		**.50**						.08
Bother eating public(73)	**.52**			.29					-.11	
Loss of sexual interest(5)	.22				.*01*					
Low energy/slow(14)	**.48**		.16		**.30**			.10		
Thoughts of ending life(15)	**.58**				**.33**					
Crying easily(20)	**.55**		-.26		.*08*					
Feeling trapped(22)	**.65**				.28	.13				
Blaming yourself(26)	**.62**	-.12			.20			-.15	-.12	
Feeling lonely(29)	**.63**		-.15		.29	-.12	-.11			.17
Feeling blue(30)	**.61**				.29	-.15				
Worrying too much(31)	**.63**	-.10			.*16*				-.16	-.17
No interested (32)	.29	.03	.20		*-*.*01*		.18		.18	
Hopeless about future(54)	**.60**				.21					
Everything is effort(71)	**.52**	-.20	.19		.*06*					
Feeling worthless(79)	**.58**		.09	.18	.15		-.09			.17
Nervousness(2)	**.55**	.12			.20	*-*.*06*	.28			
Trembling(17)	**.49**	.30				.21		.11		
Suddenly scared(23)	**.62**			-.12	.12	.*18*		.16		
Feeling fearful(33)	**.62**					.*11*	-.10	.25		
Heart pounding(39)	**.59**	.27		-.07		.*00*				
Feeling tense(57)	**.55**	.13				*-*.*16*	**.32**		-.17	
Spells of panic(72)	**.56**	.10				**.35**	.	.14		
Can’t sit still (78)	**.56**					*-*.*03*	.14			.16
Bad is happening (80)	**.63**			-.11		.*08*	.06	-.09		
Frightening thoughts(86)	**.62**				-.10	.18				.11
Easily annoyed(11)	**.43**						**.40**			-.16
Temper outbursts(24)	**.52**	-.05		-.10			**.48**			
Harm someone(63)	**.39**		.09				**.53**			
Urges to break things(67)	**.52**						**.47**	-.14		
Arguing frequently(74)	**.49**	.06					**.51**			
Shouting/throwing(81)	**.56**	.06			-.06		**.46**			.06
Afraid on the street(13)	**.45**						-.08	**.41**		-.10
Afraid to go alone(25)	**.38**							**.50**		
Afraid- transport(47)	.29	.12						**.31**		
Avoid things (50)	**.54**		.11				-.06	.29		
Uneasy in crowds(70)	**.44**			**.38**				.21		
Nervous when alone(75)	**.51**				-.15		-.06	.23		
Afraid faint in public(82)	**.45**	.25				.20		.*09*		
Others are to blame(8)	**.45**				.12		.12		.20	-.12
Can’t be trusted(18)	**.54**	-.12							.*09*	
Feeling watched(43)	**.61**			.16					.21	
Others not have beliefs(68)	**.53**	-.08					.09		.*02*	.11
Not getting credit(76)	**.52**	-.12				-.15		-.08	.*10*	.09
Taken advantage of (83)	**.59**	-.07			-.09		-.07		.*04*	
Thoughts being control(7)	**.39**					.14			.22	*-*.*06*
Hearing voices(16)	**.42**	.15				**.36**			.	.*03*
Knowing thoughts(35)	**.36**								.23	*-*.*14*
Thoughts not yours (62)	**.51**									.*05*
Feeling lonely (77)	**.62**		-.13		.16					**.40**
Sexual thoughts (84)	**.39**									.*09*
Punished for your sins(85)	**.51**	-.09			-.14					.*04*
Wrong-body(87)	**.58**	.13			-.11					.12
Never close to others(88)	**.58**									.29
Wrong-mind(90)	**.54**		.13					.09		.26

Underlined factor loadings are targeted loadings. Italic factor loadings are nonsignificant targeted items. Boldfaced factor loadings are significant at *p* < .05. For correlations, ns = not significant ((*p > .05); *p < .05; all others correlations are significant at p < .001. G = Generral; SOM = Somatization; OC = Obsessive-Compulsive; IS = Interpersonal Sensitivity; DEP = Depression; AN = Anxiety; HOS = Hostility; PANX = Phobic Anxiety; PI = Paranoid Ideation; PSY = Psychoticism.

**Table 4:** Summary of targeted and non-targeted factor loadings in the ESEM and BESEM models.

Number of	G	SO	OC	ÌS	DEP	AN	HO	PANX	PI	PY
ESEM Model										
Targeted items		12	10	9	13	10	6	7	6	10
Salient targeted items		12	7	8	9	4	6	5	0	3
Nontargeted items		71	73	74	70	73	77	76	77	73
Salient nontargeted items		3	1	2	3	0	3	0	3	3
Salient negative nontargeted items		0	0	0	0	0	0	0	0	0
BESEM Model										
Targeted items	83	12	10	9	13	10	6	7	6	10
Salient targeted items	82	10	0	3	2	1	6	3	0	1
Nontargeted items	0	71	73	74	70	73	77	76	77	73
Salient nontargeted items	-	0	0	1	0	0	0	0	0	0
Significant negative nontargeted items		0	0	0	0	0	0	0	0	0

G = Generral; S0 = Somatization; OC = Obsessive-Compulsive; IS = Interpersonal Sensitivity; DEP = Depression; AN = Anxiety; HO = Hostility; PANX = Phobic Anxiety; PI = Paranoid Ideation; PY = Psychoticism.

### Dimensions’ Ω reliabilities

In order to compute the omega coefficient reliabilities for the factors in the SCL-90-R ESEM, we used the targeted/focused factor loadings in this model. As shown (Table 5), for the ESEM model, the ω values for all factors, except PI, were above .20. ranging from .31 to .80. Furthermore, all ω except those for PI, PSY and ANX were above the adequacy threshold of .50 [71]. The SCL-90-R factor reliabilities were additionally computed using the factor loadings of the designated symptoms in the CFA 9-factor oblique model. As shown in Table 5, the ω value values (based on the CFA model) for all factors were high, ranging from .74 to .94.

**Table 5:** Inter-correlations and reliabilities of the factors in the SCL-90-R ESEM model.

	1	2	3	4	5	6	7	8	9
Somatization (1)	-	.34***	.32***	.45***	.43***	,45***	.43***	.21***	.33***
Obsessive-Compulsive (2)		-	.45***	.35***	.26***	.41***	.31***	.23***	.32***
Interpersonal Sensitivity (3)			-	.49***	.41***	.29***	.32***	.29***	.36***
Depression (4)				-	.34***	.42***	.31***	.39***	.21***
Anxiety (5)					-	.31***	.31***	.20***	.22***
Hostility (6)						-	.22***	.25***	.24***
Phobic Anxiety (7)							-	.16*	.34***
Paranoid Ideation (8)								-	.10
Psychoticism (9)									-
Reliability									
Omegas based on the ESEM 9-factor oblique model	.80	.50	.62	.62	.34	.79	.52	.16	.31
Omega (based on the CFA 9-factor oblique model	.83	.86	.79	.82	.86	.85	.94	.74	.74

### Psychopathological dimensions & family functioning aspects

Table 6 provides the standardized regression coefficients for the associations of the three main FACES IV subscales (i.e. cohesion ratio; flexibility ratio; family ratio), family satisfaction, and family communication (whilst accounting for gender), and the nine dimensions in the SCL-90-R ESEM optimum fit model. As evident for the ESEM structure (see Table 6), SOM associated negatively to family communication. DEP and PSY were also reversely associated to flexibility ratio, family satisfaction and family communication. Interestingly, ANX positively linked to flexibility ratio, family ratio, family satisfaction, and family communication. HOS was negatively associated to family cohesion ratio, family ratio, family satisfaction and family communication. Finally, O-C, PANX, and PI did not significantly tie with any of the family aspects examined. Conclusively, there were signicant external links between family functioning aspects and SOM, IS, DEP, ANX, HOS, and PSY, but not for O-C, PANX, and PI.

**Table 6:** Standardized path coefficients for the predictions of the FACES IV ratio scores for cohesion, flexibility and total scale, family communication and family satisfaction by the SCL-90-R subscale factor scores in the ESEM model.

	CR	FR	FamR	FS	FC
Somatization	-.07	-.05	-.06	-.08	-.09**
Obsessive-Compulsive	.07	-.08	-.04	.04	.06
Interpersonal Sensitivity	-.06	.05	.01	-.02	-.02
Depression	-.02	-.14*	-.11	-.25***	-.16**
Anxiety	.05	.13**	.11*	.16***	.14**
Hostility	-.11**	-.07	-.09*	-.12**	-.15***
Phobic Anxiety	-.08	-.07	-.08	.03	.01
Paranoid Ideation	-.03	-.05	-.05	-.03	-.04
Psychoticism	-.18***	-.17***	-.20***	-.19***	-.18***

CR = Cohesion Ratio; FR = Flexibility Ratio; FamR = Family Ratio; FC = Family communication; FS = Family satisfaction; ANX = attachmeent anxiety

*p < .05

**p < .01

***p < .001.

### Optimum structure selection

We selected the ESEM model with nine primary factors as our preferred model for the following reasons: (1) this structure had better global fit than all the CFA models; (2) although the ESEM and BESEM had comparable global fit indices; the ESEM patterns of loadings/cross loadings revealed better clarity. Our selection was also reinforced by the nine primary ESEM dimensions showing acceptable reliabilities. Finally, signicant associations between family functioning aspects and the SOM, IS, DEP, ANX, HOS, and PSY dimensions of the deemed as optimum model emerged.

## Discussion

The present study’s aim was twofold: a) to shed light in the controversies considering the structural dimensions of psychopathological symptoms, as assessed with the SCL-90 R and; b) to examine potential associations of specific family functioning aspects with the SCL-90 distinct psychopathological dimensions revealed. To address these aims, a large cohort of 2090 adolescents and young adults from Greece was examined. Considering the first aim, six different structural SCL-90-R models, reflecting different diagnostic conceptions were tested. First, the unidimensional CFA structure was assessed. This aligns with a transdiagnostic perspective, assuming that a general psychopathological tendency explains the different presentations [73]. Therefore, symptom specific approaches may be less effective [48]. Second, the nine-dimensional CFA structure was examined. This contents that symptom specific dimensions should be emphasized; while it contradicts the notion of a general psychopathological propensity [31,32]. Third, a higher order CFA dimensional structure with one overarching and nine direct symptom dimensions was assessed. This assumes that although different symptom dimensions may occur, to some extent these share the common influence of a general psychopathological tendency [9]. Fourth, the BCFA structure assuming one general dimension and nine symptom specific dimensions was evaluated. This structure assumes that although specific symptom dimensions apply, all (and not just part) of their common variations are attributed to a general psychopathology dimension (which therefore needs to be targeted, [47]). Fifth, the nine-dimensional ESEM was tested. This advocates the existence of distinct psychopathological dimensions, which may somehow correlate in the absence of a general psychopathological dimension [46]. Finally, the BESEM structure was evaluated. This assumes one general psychopathology dimension and nine presentation specific dimensions. These may also correlate with each other independent of the general tendency that to some extent implicates with all of them [46,47]. Adopting the theoretical structure of the SCL-90-R, the nine group/specific dimensions across all the solutions examined were SOM, O-C, IS, DEP, ANX, HOS, PANX, PI, and PSY. To conclude the best fitting structure, we appreciated four sequential steps: a) global fit; b) dimension clarity; c) dimension reliability and; d) the associations between the SCL-90 dimensions revealed were explored in relation to family functioning aspects. The fourth step of this process concurrently addressed our second aim.

### SCL-90-R dimensional structure

Considering global fitness, for all four CFA structures (unidimensional CFA, nine-dimensional CFA, higher order dimensional, and nine-dimensional BCFA), the RMSEA rates suggested good fit. In contrast, the CFI and TLI advocated poor fitness. Thus, when more than one index is considered, these structures present to have poor fit. Therefore, it is evident that, due to confirmatory procedures not allowing cross-loadings, the absence of inter-relationships between symptom specific dimensions, not accounting to a general psychopathology dimension should be excluded [43,44]. Regarding the nine-dimensional ESEM, and nine-dimensional BESEM structures tested, the RMSEA revealed good fit, and the CFI and TLI sufficient fit. Thus, both these structures were superior to the previous four (ΔCFI > .01 in all instances of pair-model comparisons). As in ESEM structures items may link to their designated specific dimensions, as well as all other dimensions [37,46], this finding confirms the occurrence of interassociations between the symptom specific dimensions, independent of the applicability of a general dimension. Furthermore, there was no significant discrepancy between the ESEM and BESEM structures in regards to ΔCFI and ΔRMSEA. Nonetheless, additional assessment of both these structures considering their dimension clarity via the use of dimensional loadings was revealing. This evaluation step suggested that the pattern of dimension loadings was clearer in the ESEM structure than the BESEM one. Finally, the ESEM structure demonstrated overall sufficient reliabilities. Given Morin and colleague’s (2015) [47] recommendation that the acceptance of a bi-factor model requires adequate reliability rates for the dimensions it proposes, the ESEM structure was chosen as the preferred structure for the SCL-90-R, at least when considering a large sample from Greece. This means that although distinct symptom dimensions may occur, in the absence of a general psychopathological tendency, these are interassociated and not completely independent to each other. This finding aligns with the numerous cross-loadings suggested in Arrindell and colleagues’ findings (2017) [9]. Furthermore, it reinforces theoretical arguments assuming that: a) psyhopathological symptoms are interrelated and; b) behavioural presentations of different diagnostic entities, such as those inquired in SCL-90, may be shared [48]. Therefore, it is likely that symptom specific interventions may also have indirect effects on non-targeted symptoms.

Our results expand the extant knowledge considering the SCL-90 dimensional structure. In contrast with previous research that has employed exclusively EFA (e,g., [38–42] and CFA (e.g., [9,12,31–35]), our work additionally assessed the dimensional structure of SCL-90-R via the employement of ESEM and BESEM structures. Thus, the results for the ESEM and BESEM for the SCL-90-R are unique. Due to this, we were able to consider alternative conceptualizations of psychopathological symptoms, that had not been previously evaluated. In that line, the theorized nine-factor oblique model for the SCL-90-R was expanded. This was achieved via the acknowledgment of interassociations between the different dimensions, irrespective of an inclusive/ general psychopathological dimension [2].

In the light of these innovations, our results appear comparative with past literature. Considering at least one global fit index suggesting sufficient fitness, past evidence has also proposed sufficient fitness for the uni-dimensional structure [12,32,33]. In addition, past CFA results have similalry revealed at least adequate fit for the nine-dimensional oblique strcuture, the second order dimensional structure and the bi-dimensional CFA structure. Nonetheless, although previous studies have favoured better global fit for the CFA bi-dimensional structure (over the CFA one-factor, nine-factor oblique and higher order factor models), this was not compared with alternative ESEM structures [9,12].

### Psychopathological dimensions and family functioning

Our results regarding the links of family effects with psychopathological presentations (as measured with the SCL-90-R) are aligning with past evidence involving young individuals [7,15–22,74]. Nevertheless, the present work methodologically expands past literature in at least two ways: a) the examination of a large and normative cohort of Greek high school students and; b) the concurrent assessment of all the nine symptom specific dimensions of the SCL-90-R and the five major family aspects, as reflected by FACES-IV. Interestingly, results highlighted that SOM associated negatively to family communication. This corresponds with evidence suggesting that unexpressed feelings and thoughts could invite psycho-somatization [16]. DEP and PSY were similalry negatively associated to family flexibility, satisfaction and communication. This result also corresponds with literature suggesting that DEP and PSY may either be exacerbated by adverse external experiences, such as those related to family functioning; or negatively impact the level of the family relationships of the carrier [25,75]. In contrast, ANX positively tied to family flexibility, satisfaction, communication and the overall family functioning (as reflected by the family ratio). This finding also entertains past literature (e.g., [76]). It may be therefore assumed, that anxiety, when not comorbid with more compromising symptoms (i.e. PSY & PI), constitutes a presentation of better-adapted individuals, likely less impacted by adverse family conditions [77]. In that line, HOS was negatively linked to family cohesion, satisfaction, communication and the overall family functioning. This also corresponds with past evidence proposing aggression as an externalized repercussion and/or a cause of poorer family conditions [7,27,78]. Nonetheless, O-C, PANX, and PI did not present significant associations with family functioning aspects in contrast with past evidence for relevant clinical populations [79,80]. This may be attributed to the nature of the sample, which (being normative) could soften links more prominent in clinical populations [81]. Overall, there were ties between specific different family functioning aspects and SOM, IS, DEP, ANX, HOS, and PSY.

### Implications & conclusion

Our results have significant theoretical/conceptual, assessment/diagnostic and prevention/intervention implications. Regarding the theoretical value of the findings, the support of the ESEM with the nine distinct synmptom dimensions as the optimum SCL-90 structure is deemed important. It implies that although distinct symptom dimensions occur, these are interassociated, while a general psychopathology factor presents not applicable. Considering the use of the SCL-90-R in assessment/ diagnosis, results do not favor the use of the general factor score. The authors of the SCL-90-R [2] proposed that this scale should be scored for the nine symptom scales (dimensions) and also the total score, as derived by the sum of all items. Furthermore, they suggested that these scores can be applied concurrently. For this suggestion to be valid, it would be required that a bi-dimensional structure should be appreciated as the optimum [71]. Two bi-dimensional SCL-90-R structures were tested (i.e. the BCFA and the BESEM) and neither was concluded as presenting the best fit. This implication is further reinforced by the rejection of the unidimensional structure. As the ESEM structure with the nine obliquely related dimensions was the preferred one, the ideal SCL-90-R scores employed in psychological assessment are those referring to the distinct symptom dimensions.

The study results also illustrate insights for family based prevention and treatment of psychopathological manifestations. In particular, specific suggestions apply given the revealed associations between SOM, DEP, ANX, HOS, and PSY with family functioning aspects. Specifically, family communication strategies should be emphasized by parents for the prevention and treatment of SOM symptoms. Similarly, increasing family flexibility, satisfaction and communication should be considered in young individuals experiencing DEP. Considering HOS and aggression, prevention and intervention protocols aiming to boost family cohesion, satisfaction and communication may also need to be emphasized. Last, family flexibility/boundaries should also be targeted for individuals presenting with PSY symptoms.

Such inittiatives might be of a particular importance when SCL-R-90 assessment is employed among adolescents and early/young adults. The majority of mental health symptoms, as well as prodromal manifestations of life-long disorders (e.g. psychosis) first present during these years (13–25, [12,13]. Thus, addressing family functioning in ways that do not exacerbate risk and promote healthy behaviours could prove crucial for young individuals’ concurrent and prospective adaptation [14].

### Limitations & further research

The major strengths of this project entail: a) the employment of a large sample; b) the application (unlike previous studies) of innovative and advanced ESEM and BESEM approaches [46] to test the structure of the SCL-90-R and; c) the comprehensive examination of all the different symptom dimensions of the SCL-90 with family functioning aspects as assessed with the FACES-IV. Despite these positives, several limitations need to be taken into account when evaluating our findings. First, it is possible that factors such as gender and ethnicity could influence ratings of SCL-90-R items. The failure to control for these effects in this study could have confounded the results. Second, as ethics approval for this study did not permit collection of information about individuals prior to inviting them to participate, there is no information about those who knew of the study but did not respond to the invitation to take part, and therefore how this affected the results. Third, as this study examined a community sample, it is uncertain if the findings are applicable to clinically diagnosed adolescents and young adults. Fourth, as the SCL-90-R and the other measures used in the study are self-report questionnaires, it is possible the ratings may have been influenced by the method used to collect them, thereby subjecting them to confounding by common method variance effects. Fifth, indirect external validities of the dimensions were not examined. Future research should include a wider range of relevant external factors, such as clinical disorders, to provide more accurate relevant testing of the external validities of the ESEM model dimensions. Notwithstanding all these limitations, our findings do support the use of the SCL-90-R in clinical practice and research. Last, they do invite for more studies in this area, that may account for the restrictions highlighted in the present work.
